# Exploring into the Unseen: Enhancing Language-Conditioned Policy Generalization with Behavioral Information

**DOI:** 10.34133/cbsystems.0084

**Published:** 2024-01-26

**Authors:** Longhui Cao, Chao Wang, Juntong Qi, Yan Peng

**Affiliations:** ^1^ School of Future Technology, Shanghai University, Shanghai, China.; ^2^ Institute of Artificial Intelligence, Shanghai University, Shanghai, China.

## Abstract

Generalizing policies learned by agents in known environments to unseen domains is an essential challenge in advancing the development of reinforcement learning. Lately, language-conditioned policies have underscored the pivotal role of linguistic information in the context of cross-environments. Integrating both environmental and textual information into the observation space enables agents to accomplish similar tasks across different scenarios. However, for entities with varying forms of motion but the same name present in observations (e.g., immovable mage and fleeing mage), existing methods are unable to learn the motion information the entities possess well. They face the problem of ambiguity caused by motion. In order to tackle this challenge, we propose the entity mapper with multi-modal attention based on behavior prediction (EMMA-BBP) framework, comprising modules for predicting motion behavior and text matching. The behavioral prediction module is used to determine the motion information of the entities present in the environment to eliminate the semantic ambiguity of the motion information. The role of the text-matching module is to match the text given in the environment with the information about the entity’s behavior under observation, thus eliminating false textual information. EMMA-BBP has been tested in the demanding environment of MESSENGER, doubling the generalization ability of EMMA.

## Introduction

Reinforcement learning (RL) has demonstrated its effectiveness in multiple domains by continuously trial-and-error searching for optimal policies [1,2,3]. However, in reality, it usually does not allow agents to repeatedly try in task scenarios, which may waste a lot of resources [4]. In addition, executing tasks only in the same scenario limits the application of RL methods. In order to solve these problems, it is necessary to improve the generalization ability of RL policies so that agents can be trained in similar environments and applied to unseen scenarios. To showcase the capacity for generalization within RL policies, agents need to acquire policies within analogous settings and eventually employ them in unseen situations. However, in most cases, even when there is little variation between the training and unseen environments, policies generated solely by RL methods still struggle to handle challenges encountered in similar but complex situations due to the agent’s extensive exploration within a single environment [5,6]. In order to improve the generalization of RL policies, agents need to be able to delve deeper into the more complex connections between the learning environment and observations.

One of the reasons people can quickly adapt to unseen environments is because they have previously learned how to take appropriate actions in similar situations they might encounter in the unseen environment [7]. The corresponding connections between these scenarios and actions can be provided to agents through textual information. Many researchers, inspired by human behavior, attempt to combine observation space with textual information, enabling agents to learn textual content in similar environments and apply policies to unseen environments. This method of combining text information with environmental observation is called language-conditioned policy, which successfully promotes the application of natural language processing technology in RL. The core of this policy is to learn the correlation between text and observations, thereby helping to extend RL policies to unknown environments.

Recently, Zhong et al. [8] and Hanjie et al. [9] successfully introduce RL methods into language information under text conditions, enabling them to be applied in unseen environments. This language-conditioned policy enables agents to understand better the relationship between textual information and the observed environment, thereby improving the generalization ability of the policy. The main difference between language-conditioned policies and other RL policies is that they take both world observations and text manuals as input and output actions accordingly. The challenge in language-conditioned policies is enabling agents to learn and connect textual information to world observations. Textual descriptions allow the agent to solve similar tasks in unseen environments.

In order to establish task scenarios for language-conditioned policies and verify the effectiveness of introducing text information, Hanjie et al. [9] propose MESSENGER in their research. The training and validation environment of MESSENGER includes a series of similar but different tasks. The challenge for MESSENGER is to require agents to be able to understand the relationship between text descriptions and entity symbols during the training process. When the task environment changes, the agent needs to be able to execute the task based on any given text description, even if there are inconsistencies in the description or changes in the location or name of entities. This environment encourages agents to effectively utilize textual information to infer and apply policies to adapt to different situational changes.

MESSENGER is an interesting research platform that helps explore the generalization and reasoning abilities of language-conditioned policies in RL. It has 3 training stages with gradually increasing difficulty levels. The condition for winning in all stages is to avoid the observed enemy and obtain a message based on the text description, ultimately converging with the target entity.

1. In the first stage, there are 3 types of entities. Each entity represents only one type of description (e.g., queen for goal, ball for enemy, and plane for messenger).

2. In the second stage, descriptions of the movement of the entities are added (e.g., immovable queen, chase ball, and fleeing plane). It is worth noting that each entity name is unique, despite the addition of a motion description.

3. The text description and entity behavior in the third stage are similar to those in the second stage. The difference is that there are 6 texts and 5 entities. This means that not every text has a corresponding entity. In the text description, there are identical entities with different patterns of motion.

**Fig. 1.**
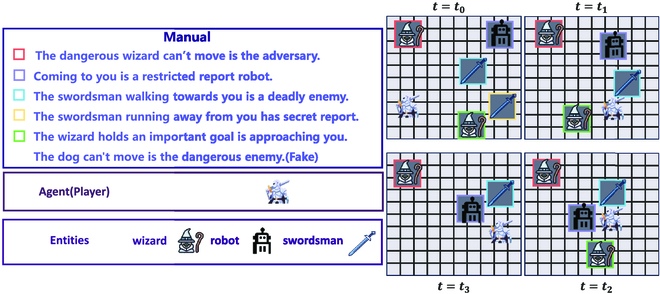
Observation space for the third stage of MESSENGER. The manual of the map is listed on the left side of the figure. There are 3 types of entities (i.e., mage, sword, and robot) with the agent (i.e., knight) in the graph.

The entity behavior and text of MESSENGER in the third stage contain all the content of the first 2 stages. The observation of MESSENGER in the third stage is shown in Fig. 1. The agent’s task is to find the entity with the information based on the text description and finally reach the location where the target entity is located. The game begins at *t* = *t*_0_, with the agent interacting with the entity that holds the information (i.e., swordsman) and gathering it at *t* = *t*_1_. At *t* = *t*_2_, the agent makes an effort to meet with the target while avoiding enemies. Finally, at *t* = *t*_3_, the agent successfully interacts with the target (i.e., wizard) and completes the episode.

Hanjie et al. [9] propose the EMMA framework for the test environment MESSENGER. This framework successfully generalizes the policies learned during training to an unseen environment by learning the correspondence between text and entity symbols.

**Existing issues.** Nevertheless, there are certain issues within the entity mapper with multi-modal attention (EMMA):

• **Absence of behavioral information.** To the EMMA framework, the main focus is on the relationship between entity names and text, with insufficient consideration of behavioral information in the text. This may result in inadequate handling of entities in text descriptions that share the same name but have different motion behaviors (e.g., immovable mage and fleeing mage). This limitation could potentially impact the generalization capability of EMMA, especially in situations where it is necessary to distinguish entities with the same name but different motion behaviors.

• **Existence of interference information.** As for the MESSENGER environment, the presence of interference information may affect the agent’s policy selection, and the EMMA framework is currently unable to eliminate or process this interference information effectively. This situation may cause unnecessary interference to the agent while executing tasks, reducing its performance and decision-making accuracy.

To address the above issues, we propose the entity mapper with multi-modal attention based on behavior prediction (EMMA-BBP). The framework improves the generalization performance of language-conditioned policies in unseen environments through 2 components. Regarding issue 1, we propose a behavioral prediction module to enhance the model’s ability to distinguish based on behavior. In this module, agents may predict the behavior of individual entities using a behavioral prediction algorithm. As a result, the agent adds detailed descriptions of the behavior of each entity. Entities are changed from the definition of their name (e.g., ball, queen, and plane) to the description of their behavior (e.g., chase ball, fleeing plane, and immovable queen). Regarding input text, the third stage of MESSENGER inputs more textual information (6 pieces of textual information) than entities in the observed environment (5 pieces of textual information). To address issue 2, we propose a text-matching module to reduce the effect of fake information in the unseen environment. Five messages with the most similar similarity were obtained by doing text matching with the original manual by the entity descriptions with behaviors obtained in the first part.

**Contributions.** In summary, the main contributions of our work are as follows:

• We propose the EMMA-BBP framework, which comprises 2 essential components: a behavioral prediction module and a text-matching module. With EMMA-BBP, agents acquire behavioral insights from textual information, effectively resolving ambiguities arising from diverse behaviors. Simultaneously, our framework substantially bolsters policy generalization while efficiently eliminating interference information.

• We generate the corresponding text-matching dataset based on the text description of MESSENGER. This dataset has effectively facilitated the training of our text-matching module.

• We have conducted a series of experiments in the MESSENGER environment, conclusively illustrating that EMMA-BBP’s generalization capacity has doubled compared to EMMA.

**Paper organization.** We provide the paper organization as follows: The “Related Work” section introduces the related work of this paper. The “Background” section provides background knowledge on the methods and experiments presented in this paper. The “Method” section presents the structure and methods of our EMMA-BBP framework. The “Experiments” section presents our experimental method based on 4 baseline models. In the “Limitation and Discussion” section, we present the current limitations of the model and discuss our main insights. Finally, the conclusion of this paper and future work are summarized in the “Conclusion and Future Work” section.

## Related Work

### Language-conditioned RL

The reason why humans can quickly adapt to unseen environments is that they are able to learn abstract concepts through textual information and apply them to corresponding situations [10]. Inspired by this, many studies have explored the learning of manual by making agents for solving tasks in similar environments. Chaudhury et al. [11] proposed NESTA, a modular symbolic text agent, in the context of text-based games (TBGs). NESTA successfully converted observation text descriptions into ternary form for agents to learn. For complex multitasking problems, Chen et al. [10] proposed to use natural language instructions generated by a neural network as a high-level representation of subtasks and to formulate policies for achieving the goal conditions given these instructions. Pang et al. [12] proposed to replace natural language with task language to improve agent’s understanding of human language. Wu et al. [13] improved the speed of RL to train policies in the form of auxiliary rewards generated by reading game instructions. Zhong et al. [14] unified the currently existing RL language interaction environments and proposed the Symbolic Interactive Reade (SIR) framework, which performs better in multiple language navigation tasks in an integrated manner. Ding et al. [15] extended RTFM and MESSENGER to multi-agent scenarios and proposed the EnDi framework to address language-conditioned policies problems in multi-agent systems. Cao et al. [16] proposed a reward shaping method based on natural language, which helps agents achieve target states faster by utilizing natural language instructions. Mu et al. [17] used natural language as a medium to highlight abstract concepts in the environment and solved the difficulty of sparse rewards in RL through natural language. Li et al. [18] believe that the entanglement between semantic information and specific task state information hinders the learning of semantic invariance and reusable representations. Therefore, Li et al. proposed an element randomization method that successfully extracted task-related but environment-independent semantic information from instructions using a set of environments with randomized elements.

In previous work, agents were trained to complete assigned tasks by comprehending written information. Although this approach helps apply RL to different scenarios, current models struggle with uncertainties arising from entities displaying multiple behaviors. Therefore, it is necessary to provide agents with enough behavioral information to improve their performance.

### Semantic textual similarity

In natural language processing, judging semantic similarity is a basic task. The traditional text-matching algorithm term frequency-inverse document frequency (TF-IDF) compares the similarity of texts by calculating word frequencies. However, for mining complex semantic relationships between texts, neural network-based text-matching algorithms perform better [19,20,21]. There are several classical neural network text-matching algorithms available, such as DSSM [22], CDSSN [23], and LSTM-DSSM [24]. Since the release of BERT [25], pretrained language models have gained popularity due to their outstanding performance. Lyu et al. [26] proposed language knowledge enhancement graph transformer (LET) to address the problem of synonym ambiguity in Chinese words. Li et al. [27] and Su et al. [28] solved the problem of original text matching via postprocessing sentence embedding to solve the problem of high similarity that sentence pairs have due to the original BERT. Jiang et al. [29] proposed a new contrastive learning method PromptBERT to better learn the representations in sentences.

While certain models may excel in certain situations, our application requires a fast and precise comparison of the 2 texts. Additionally, the goal of text matching is to avoid a large number of pretraining processes by minimizing the training parameters of the model and making more effective use of time and resources.

## Background

In this section, we will provide a brief overview of background knowledge pertaining to the incorporation of language-conditioned policies. The key distinction between language-conditioned policies and other RL policies lies in their input, which encompasses not only the observation space but also textual information. The text description of language-conditioned policies includes an introduction to entity names, types, and other content. The agents acquire dynamic information from the text to determine the optimal policy, denoted as *π*, aimed at maximizing the cumulative reward *R_π_*(*O*, *t*), where *O* represents the observation map and *t* corresponds to the textual description of the environment.

### Observation map

The observation map $O\in {\mathbb{R}}^{h\times w\times d}$ includes global entity location information, where *h* represents the map’s height, *w* represents the map’s width, and *d* denotes the embedding of entity learning. In the third stage of MESSENGER, the map is taken as a 10 × 10 grid environment.

Since the entities in the grid environment have movement information, each entity’s movement in a round may choose to move up, down, left, and right by one frame or stay still. Therefore, the observation map needs to be synchronized to update the position information of the other entities after moving one step by the agent. In the map, agents may interact with other entities, yet other entities cannot interact with each other. In the third stage of MESSENGER, AO means that the agent does not carry information at present and will interact with entities that have information. When the agent coincides with the coordinate position of the entity containing information, it changes to AM with information and gets some reward. When the agent (AO/AM) coincides with an enemy coordinate position, it dies, fails in this game episode, and receives a penalty. When the agent (AO/AM) coincides with the target coordinate position, it will obtain the game win and end the episode. The agent’s observation map is depicted in Fig. 1. In addition to the entity’s positional coordinates featured on the observation map, it also incorporates the learned embedding information denoted as *d*.

### Text manual

The manual contains descriptions of entity names, task types, and motion types, as depicted in Fig. 1. The text manual remains consistent across game episodes yet varies between games. The description of the same entity varies within each episode. For instance, an entity named as plane might be described as jet, aircraft, airplane, and so forth in different episodes. The model is trained using various descriptions of the same entity, obtained from Amazon Mechanical Turk [30], to improve its understanding of semantically similar sentences.

## Method

### Problem definition

**Input.** Within our framework, it is imperative to furnish the agents with a manual *t* containing entity descriptions and a global observation map *O* As shown in Fig. 1.

**Output.** Our objective is to task the agents with the task of removing inaccurate information from the initial manual *t* based on entities’ behavior, thereby producing a revised manual *t*^′^ devoid of interference information. Simultaneously, according to the description of the new manual *t*^′^ and information from the observation map *O*, we aim to generate the optimal policy *π* to maximize the ultimate reward.

**Example.** For instance, in the scenario depicted in Fig. 1, the manual *t*^′^ that remains following the removal of interference information encompasses the following: (a) The dangerous wizard can’t move is the adversary. (b) Coming to you is a restricted report robot. (c) The swordsman walking towards you is a deadly enemy. (d) The swordsman running away from you has secret report. (e) The wizard holds an important goal is approaching you. The optimal policy obtained by the agent based on the observation map *O* and new manual *t*′ is, first, move to the right to merge with the information entity (yellow boxed swordsman) (i.e., *t* = *t*_1_). Then, avoid the enemy (green boxed wizard, purple boxed robot) and find a route to merge with the target (i.e., *t* = *t*_2_). Finally, merge with the target entity (wizard in the green box) (i.e., *t* = *t*_3_).

**Fig. 2.**
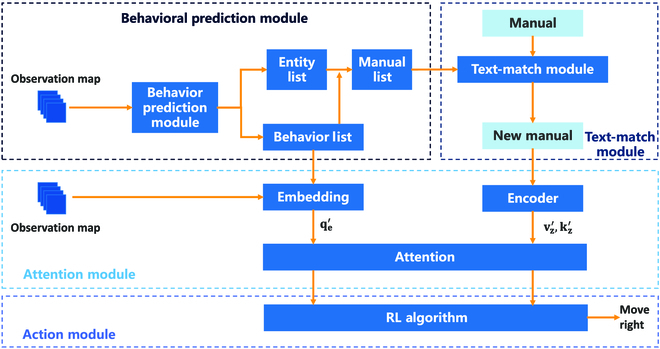
EMMA-BBP framework. Our framework mainly consists of a behavioral prediction module, a text-matching module, and an RL algorithm combined with the Attention module. Behavioral prediction module uses a prediction algorithm to determine how an entity will behave. The text-matching module helps to remove descriptions of entities that do not exist. Once the Attention module processes the entity mapping and text, it provides an observation value to the RL algorithm, which outputs agent actions.

Next, we will center on the structure of the EMMA-BBP framework. In EMMA-BBP, the agent predicts and labels the movement behavior of entities to learn their behavioral information. In order to reduce the impact of false text in the third stage of MESSENGER, a text-matching module is introduced, aiming to select the 5 texts with the highest similarity to the description of entity behavior. The agent learns the deep connection between entities and texts on the map by processing new textual information and entities’ behavior with the Attention module. Once the Attention module has processed it, the information is observed by the Action module to generate the best possible action. The EMMA-BBP system comprises 4 components: the behavioral prediction module (“Behavioral prediction module” section), text-matching module (“Text-matching module” section), attention module (“Attention module” section), and action module (“Action module” section). Figure 2 presents the EMMA-BBP framework.

### Behavioral prediction module

**Fig. 3.**
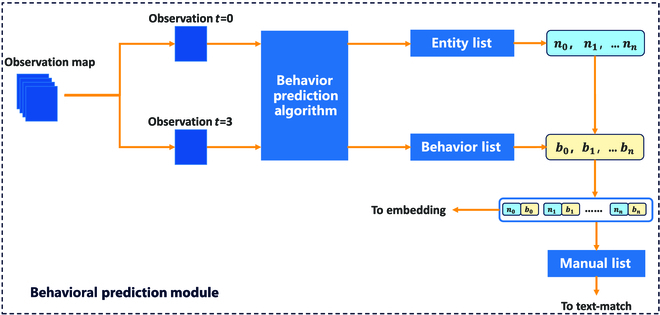
Behavioral prediction module. To generate a new manual, the agent analyzes the observation maps from 4 rounds of its activity. The agent obtains a list of entity behaviors and names by comparing the coordinate relationship between the beginning (i.e., Observation *t* = 0) and end (i.e., Observation *t* = 3) of each round. These are then combined to create the manual.

The behavioral prediction module refers to the way humans judge action behavior. Figure 3 illustrates the structure of the behavioral prediction module.

In order to determine the location of the entity in relation to the agent, the agents come to a stop for 4 rounds. The observation map from those 4 rounds is then used as input to predict the entity’s movement based on the coordinates from the fourth and first rounds. The behavioral prediction algorithm is outlined in Algorithm 1.

Utilizing the observation map, we derive a comprehensive list of entity coordinate information:

$$C_{t=0}=\left[c_{0},c_{1},c_{2},c_{3},c_{4}\right],C_{t=3}=\left[c_{0}^{\prime },c_{1}^{\prime },c_{2}^{\prime },c_{3}^{\prime },c_{4}^{\prime }\right].$$ (1)

It is important to note that the coordinate list recorded here is based on the top-down coordinates of the entities in the observation map. Thus, the same entity may have a different position in *C*_*t*=0_ than in *C*_*t*=3_, making it challenging to predict entity behavior. Hence, it is necessary to adjust the sequence of entity coordinates in the list *C*_*t*=0_.

Due to the limitations of the environment, the entities change at most one coordinate by one frame per turn. Given the vast shift in coordinates due to changes in entity order, we consider leveraging this to detect potential alterations in the order of entities between *C*_*t*=0_ and *C*_*t*=3_.

$$|c_{\mathrm{ix}}-c_{\mathrm{ix}}^{\prime }+c_{\mathrm{iy}}-c_{\mathrm{iy}}^{\prime }|\left\{\begin{array}{cc} \leq 2, & \;\text{Pass}\\ \geq 2, & \;\text{ChangeOrder} \end{array}\right.$$ (2)

where *c_ix_*, *c_iy_* ∈ *C*_*t* = 0_ and $c_{\mathrm{ix}}^{\prime },c_{\mathrm{iy}}^{\prime }\in C_{t=3}$. When the order of entity coordinates changes due to the order of records, the position of the coordinates in *C*_*t*=0_ needs to be changed to obtain $C_{t=0}^{\prime }$, which in turn causes entity in $C_{t=0}^{\prime }$, and the entities in *C*_*t*=3_ correspond to each other.

Based on the relationship between the coordinates in $C_{t=0}^{\prime }$ and *C*_*t*=3_, the agent obtains a set of lists about the behavior of entities by Algorithm 1.

$$B=\left[b_{0},b_{1},b_{2},b_{3},b_{4}\right].$$ (3)

Simultaneously, based on the recording of the entities in the observation map, the agent obtains a list of recorded entity names:

$$N=\left[n_{0},n_{1},n_{2},n_{3},n_{4}\right].$$ (4)

According to the behavior list *B* and name list *N* in the behavioral prediction module, the agent obtains the corresponding entity name and behavior. Simultaneously, the entity description is updated through the behavior list so that the entity description contains behavior information. Next, the text describing the motion of the entity is sent to the text-matching module for text matching with the original manual.


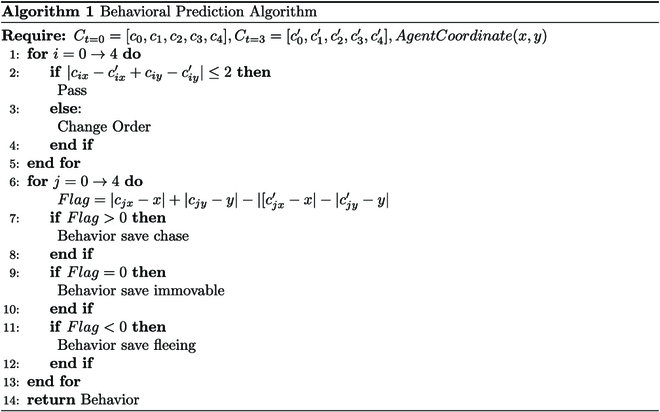


### Text-matching module

**Fig. 4.**
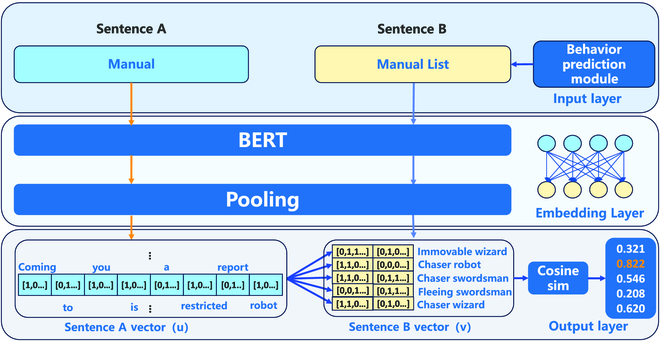
CoSENT framework in inference. First, the new manual and original manual obtained from the behavioral prediction module are encoded separately through BERT. Their sentence vectors *u* and *v* are obtained through the pooling layer, respectively. Finally, the cosine similarity between *u* and *v* is calculated to obtain the approximate relationship between the 2 sentences.

We choose the cosine sentence (CoSENT) [31] model for the text-matching part. CoSENT improves on the scheme of Sentence-BERT [32] for sentence vectors of cosine rank loss. In EMMA-BBP, the entity motion text derived from the behavioral prediction module only contains the entity’s name with the motion behavior and does not include the definition for the entity (e.g., goal, enemy, and messenger). Therefore, in order to understand more about the description of entities in the text, it is still necessary to feed the text information generated by the behavioral prediction module into CoSENT to process the original information. By using the text-matching module, the top-5 similar are selected as new texts to be entered into EMMA. The framework of the CoSENT model is shown in Fig. 4.

In calculating the similarity between sentence *S_A_* and sentence *S_B_* statements, we adopt the cosine similarity:

$$\text{Similarity}=\frac{\langle S_{A},S_{B}\rangle }{\left\|S_{A}\|\times \|S_{B}\right\|},$$ (5)

where 〈*S_A_*, *S_B_*〉 denotes the dot product of vectors, and ‖*S_A_*‖, ‖*S_B_*‖ denote the paradigms of vectors:

$$\|S_{A}\|=\sqrt{a_{0}^{2}+a_{1}^{2}+\ldots +a_{n}^{2}},$$ (6)

$$\|S_{B}\|=\sqrt{b_{0}^{2}+b_{1}^{2}+\ldots +b_{n}^{2}},$$ (7)

For the loss function, we use cross entropy loss (CEL) [33] as shown in the following equation:

$$\begin{array}{c} \text{Loss}=\frac{1}{N}\sum_{i}L_{i}=\frac{1}{N}\sum_{i}-\left[{\text{label}}_{i}\times \text{log}\left(p_{i}\right)+\left(1-{\text{label}}_{i}\right)\times \text{log}\left(1-p_{i}\right)\right], \end{array}$$ (8)

where *label_i_* denotes the label of sample *i*. When 2 sentences resemble, it will be marked as “1.” Otherwise, it will be marked as “0.” *p_i_* denotes the probability that sample *i* is predicted to be similar. *N* represents the number of samples.

### Attention module

**Text encoder.** In the text embedding stage, we utilize the EMMA approach. However, we implement the entity description $z^{\prime }$ from the CoSENT model, which removes erroneous textual information and contains only 5 texts.

For description $z^{\prime }$, we use the BERT-based model to segment and encode descriptions to generate tokens *t*_1_, *t*_2_, ……*t_n_*. Then, referring to the content in the Attention module [34], we process the generated tokens. We generate corresponding value vectors $v_{z^{\prime }}$ and key vectors $k_{z^{\prime }}$ based on equations:

$$k_{z^{\prime }}=\sum_{i=1}^{n}\alpha_{i}W_{k}t_{i}+b_{k},\alpha =\text{Softmax}\left({\left(u_{k}.t_{j}\right)}_{j=1}^{n}\right)$$ (9)

$$\begin{array}{c} u_{z^{\prime }}=\sum_{i=1}^{n}\beta {\mathrm{iW}}_{v}t_{i}+b_{u'}\beta =\text{Softmax}\left({\left(u_{u}.t_{j}\right)}_{j=1}^{n}\right) \end{array}$$ (10)

where *W_k_* and *W_v_* are the feature vectors and *b_k_* and *b_v_* are the deviations. *W_k_*, *b_k_*, *u_k_*, *W_v_*, *b_v_*, and *u_v_* are the learning parameters. During the training policies process, the learned parameters are used to focus on the relationship between entity descriptions and textual information [25,35].

**Entity embedding.** To enable the entities to correspond to the textual information, embedding is performed on each entity to generate the query vector *q_e_* of dimension *d*. However, it is worth noting that before embedding, the original entity symbols need to be processed. That is, the behavioral information generated in the behavioral prediction module needs to be added to the original entity symbols. The vector for the entity embedding can be expressed as:

$$q_{e}^{\prime }=q_{\text{name}+\text{behavior}},$$ (11)

Then, we use the scaled dot product attention to obtain a representation of the entity that contains behavioral information as $X_{e}^{\prime }$:

$$X_{e}^{\prime }=\sum_{z\subset Z^{\prime }}\text{Soft}{\text{max}}_{z^{\prime }}\left(\frac{q_{e}^{\prime }.k_{z}^{\prime }}{\sqrt{d}}\right)u_{z}^{\prime },$$ (12)

where *d* represents the learning embedding dimension of the entities. Entities are associated with text descriptions through $k_{z}^{\prime }$, $q_{e}^{\prime }$, and $v_{z}^{\prime }$ in the Attention module. In this context, $k_{z}^{\prime }$ and $q_{e}^{\prime }$ are focused on the entity described in the text, such as fleeing ball, while $v_{z}^{\prime }$ is focused on the entity’s definition, like goal. Then, we put the resulting $X_{e}^{\prime }$ into the tensor $X\in {\mathbb{R}}^{h\times w\times d}$ , where $X_{e}^{\prime }$ is in the same position as the entity is in observation map.

### Action module

First, to obtain the distribution of the action *π*(*a*| *o*, *Z*), we perform a 2-dimensional convolution operation on $X_{e}^{\prime }$ in both the *h* and *w* dimensions, resulting in the generation of features denoted as *y*. $X_{e}^{\prime }$ represents the text-conditioned representation of each entity on the map. In the MESSENGER environment, the actions that agents may take are discrete. Therefore, in order to obtain the actions of the agents, we process feature *y*. Subsequently, we flatten the features *y* to obtain $y^{\prime }$. Last, $y^{\prime }$ is fed into a fully connected feedforward network (FFN) [36], which utilizes the Softmax function to derive possible actions.

$$\left\{\begin{array}{cc} & y=\text{Conv}2D\left(X_{e}^{\prime }\right)\\ & y^{\prime }=\text{Flatten}\left(y\right)\\ & \pi \left(a|o,Z\right)=\text{Softmax}\left(y^{\prime }\right) \end{array}\right.$$ (13)

In addition, for the trade-off between exploration and exploitation, agents have a 0.05 chance of taking random actions when selecting each action.

## Experiments

### Experiment setup

**Dataset.** Our text-matching module operates as a supervised model. We generate labeled text pairs from the entity behavior and environment manual generated by the behavioral prediction module. The generated training set size is 3,619 text pairs. The test set size is 2,096 text pairs. The size of the verification set is 2,097 text pairs.

**Baseline.** To evaluate whether our framework and modules have better generalization performance, we compare the following baseline models:

• **EMMA [9]:** EMMA is the basic framework of EMMA-BBP. In this framework, all content of EMMA is preserved. For EMMA, a total of 6 text messages are entered. Five of them describe entities’ information, while the remaining one describes content that does not match any entities’ information. EMMA associates entity names with textual descriptions through the Attention module. In EMMA, learning the behaviors of entities is achieved by introducing buffers.

• **EMMA in Less-manual (Ideal):** EMMA in less-manual (Ideal) framework. In order to test the effectiveness of removing confusing information, we introduce an idealized text-matching module. In this framework, only 5 messages are used after removing confusing information, instead of the initial 6 messages used in EMMA. Besides, the remaining parts of it remain the same as Hanjie et al. [9].

The above 2 baseline models are used to compare EMMA and its variant, which demonstrates that removing confusing information helps improve the success rate of agents, especially in unseen environments.

• **EMMA-BBP in All-manual (Without Text-matching Module):** EMMA-BBP in All-manual framework. To further verify the effectiveness of the behavioral prediction module, in this framework, we remove the text-match module from EMMA-BBP and keep only the behavioral prediction module. In this framework, we input 6 messages with confusing information.

• **EMMA-BBP in Less-manual (Ideal):** To compare the gap between our text-matching module and the ideal situation, we introduced EMMA-BBP in Less-manual (Ideal). This framework is similar to EMMA in Less-manual. However, unlike EMMA in Less-manual, the EMMA-BBP in Less-manual adds an additional behavioral prediction module. In this framework, instead of using the text-matching module to eliminate the confusing information, we remove it directly from the environment.

The above 2 baseline models are variants of EMMA-BBP. The purpose is to demonstrate the importance of introducing behavioral prediction in EMMA-BBP and to analyze the ideal situation of EMMA-BBP.

**Metrics.** For different experimental stages, we use the following metrics.

• **Text matching: Success rate and Spearman coefficient.** In the first part of the experiment, we evaluate the success rate of our text-matching model.

$$s=\frac{C_{\mathrm{win}}}{C_{\mathrm{win}}+C_{\text{loss}}},$$ (14)

In Eq. 14, *C*_win_ represents the total number of times the text-matching model correctly selects 5 text messages. *C*_loss_ represents the total number of times 5 text messages were not correctly selected, and *s* represents the success rate.

In training CoSENT, the Spearman coefficient [37] is used as a result evaluation metric. Spearman is used as a parameter for comparing the correlation of 2 datasets and for evaluating the monotonic relationship between 2 datasets. The calculation of the Spearman coefficient during our training process is as follows:

$$r=\frac{\sum ‍\left(R_{x_{i}}-{\bar{R}}_{x}\right)\left(R_{y_{i}}-{\bar{R}}_{y}\right)}{\sqrt{\sum ‍{\left(R_{x_{i}}-{\bar{R}}_{x}\right)}^{2}\sum ‍{\left(R_{y_{i}}-{\bar{R}}_{y}\right)}^{2}}},$$ (15)

where *R_x_i__* and *R_y_i__* denote the order of the *i*th *x*-variable and *y*-variable after sorting, respectively, and ${\bar{R}}_{x}$ and ${\bar{R}}_{y}$ denote the mean values of *R_x_i__* and *R_y_i__*, respectively. In our experiment, *x* denotes the labels in the val set and *y* denotes the value of the predicted cosine similarity.

• **Generalization performance: Winning rate and Reward value.** In the second part of the experiment, the generalization performance of EMMA-BBP is compared with that of several baseline models. In this part, we evaluate the effectiveness of the models by the win rate and the average value of rewards, where win rate is the number of episodes in which the agent successfully converges with the target entity. The agent’s performance determines the reward. If the agent is able to obtain the information successfully, it is recorded as “+0.5.” If the agent is able to rendezvous with the target successfully, it is recorded as “+1.0.” Otherwise, the set is registered as a failure and receives a reward of “−1.0.”

**Implementation and training details.** We train our model on a computer with an NVIDIA 2080Ti with 11 GB of RAM and a 12-core CPU. It takes 6 days to complete the training of stage 3, and a total of 1.525 × 10^6^ episodes are trained. Proximal policy optimization [38] and Adam optimizer [39] are used to train these models. We set the learning rate to 5 × 10^−5^ and limit the maximum step size of each set of exploration to 128 steps, with “−1.0” reward if the agent does not complete the goal within the specified step size.

### Experimental results

In this section, we present the training results of the text-match module and demonstrate the training process of the models. In order to verify that the removal of confusing false information in manual facilitates the generalization of the models, at the end of this section, we show the performance effect of all the models in the third stage of MESSENGER in the test set and validation set. In summary, our EMMA-BBP performs better than EMMA.

**Fig. 5.**
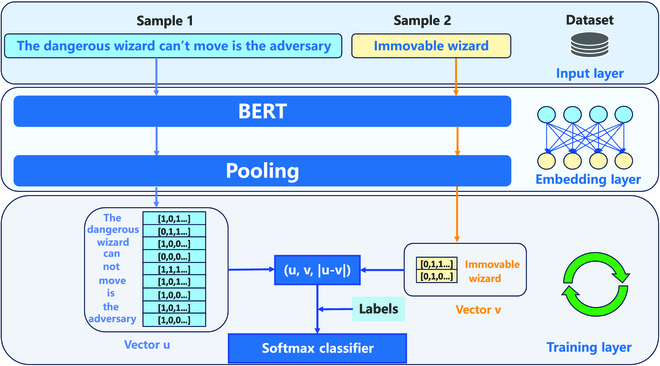
CoSENT in training. Unlike the inference process, during training, we need to provide each utterance pair with the correct label for the network to learn. CoSENT training aims to make the utterance vectors produced by similar sentences after passing through the BERT embedding and the pooling layer have a smaller distance.

**Text matching.** In the CoSENT training phase, the MESSENGER manual served as the dataset. The behavioral prediction module’s potential output statements are considered sample 1, while the manual’s statements are considered sample 2. Subsequently, after encoding by BERT, the vector *u* of sample 1 and the vector *v* of sample 2 are obtained. The values of *u*, *v*, and ∣*u* − *v*∣ are combined to create a set of feature vectors, where ∣*u* − *v*∣ is the vector formed by taking the absolute value of each element of *u-v*. During training, the model is also fed with pre-made label values *Labels*. The above 3 vectors are sent into the fully connected layer and a 2-class task is performed, as shown in Fig. 5.

**Fig. 6.**
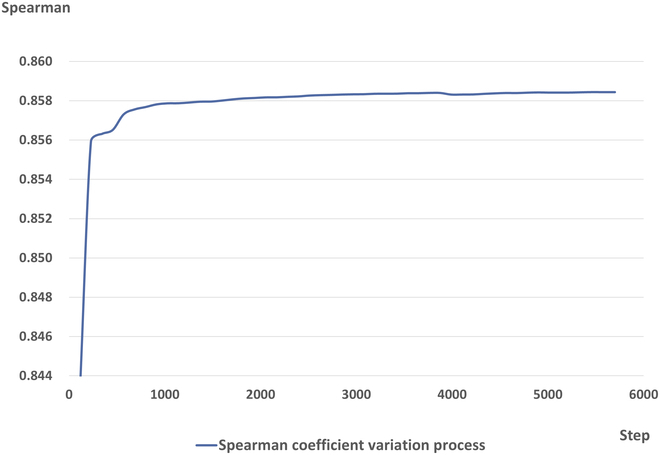
Spearman correlation analysis. The blue line shows the variation of the Spearman coefficient with the number of iteration steps. Our experiment iterates for 5,700 steps, and the final Spearman coefficient is 0.858.

During the training process, the Spearman coefficient gradually increases with the number of iterations. We show the training results of CoSENT, as shown in Fig. 6. CoSENT, a text-matching module, quickly converges with a final loss function value of 0.0014 after 100 steps, as shown in the graph. In addition, we separately extract CoSENT from the behavior module to test whether the provided new manual can successfully eliminate error information in the training set, test set, and validation set of the third stage of MESSENGER.

We randomly select 1,000 games from the training, testing, and validation sets. The success rate is calculated if CoSENT eliminates confusing information. Otherwise, it is marked as a failure. Table 1 shows the measurement results.

**Table 1.** Text-matching module performance

Module	Train↑	Test↑	Val↑
Text-matching module	0.691	0.626	0.637

As shown in Table 1, we find that the performance of the text-matching module is not as satisfactory as what we expect. To verify the correctness of text matching, we separately extract the text-matching module and conduct the following experiments. We use the dataset we have created to verify the success rate of text matching. In our experiment, if the absolute difference in similarity between 2 sentences is less than “0.3,” it is considered a successful match. The obtained data are shown in Table 2.

**Table 2.** The success rate of text matching

Dataset	Number of successes	Total	Success rate↑
Train	3,576	3,619	0.988
Test	1,967	2,096	0.938
Val	1,980	2,097	0.944

From our text-matching experiment, we deduce the following noteworthy conclusions: (a) CoSENT, as a text-matching model, exhibits rapid convergence and high precision and demands minimal training time. (b) The main reason for the error in the text-matching task is the failure of the behavioral prediction module to predict the behavior. Although the entity behavior is defined as chase or fleeing in MESSENGER, the entity did not make the corresponding action in 4 rounds, which would trick the behavioral prediction module into making wrong predictions.

**Fig. 7.**
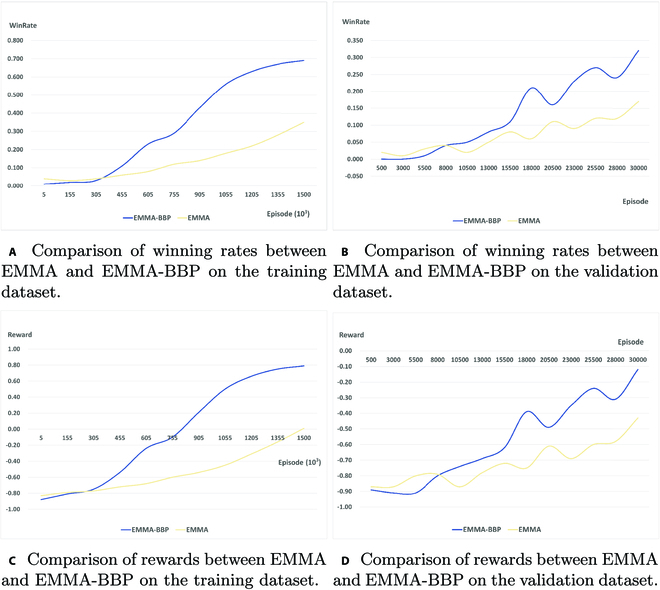
Comparison of the performance of EMMA-BBP and EMMA during the training process. (A) Change in win rate over the training dataset during training. (B) Change in win rate on the validation dataset during training. (C) Change in reward on the training dataset during training. (D) Change in reward on the validation dataset during the training.

**Generalization.** Figure 7 displays the winning rates and rewards for EMMA-BBP and EMMA on the early training and validation sets.

In EMMA-BBP training, we train a total of 1.565 × 10^5^ episodes. The blue line shows EMMA-BBP’s winning rate and reward changes on the training and testing sets, while the yellow line shows EMMA’s winning rate and reward changes on the training and validation sets. As the training episode increases, the win rate and reward of EMMA-BBP also increase. During the training episode, the win rate reaches 70% and rewards 0.8. However, during the validation episode, the win rate drops to 30% and rewards −0.1. The cause of this issue is connected to the configuration of the MESSENGER environment. Although the agent made judgments about the entities’ behavior, the decisions’ results were still largely dependent on the movement of the entities during the 4 rounds at the beginning. If the entity’s motion during the 4 rounds does not match the textual description, it will lead the agent to label the entity with the wrong behavior, affecting the training results to some extent.

We compare the EMMA-BBP with the baseline model on the training, testing, and validation sets. The testing scheme is to validate each framework 10 times on 1,000 episodes (10,000 episodes in total), and the win rate of these 10 times is averaged and recorded. The test results are shown in Table 3.

**Table 3.** Performance comparison between EMMA-BBP and baseline models

Module	Train↑	Test↑	Val↑
EMMA [9]	0.3161	0.1380	0.1409
EMMA in Less-manual	0.3373 (+0.0212)	0.1743 (+0.0363)	0.1823 (+0.0414)
EMMA-BBP in All-manual	0.6781 (+0.3620)	0.2752 (+0.1372)	0.2806 (+0.1397)
EMMA-BBP(Ours)	0.6124 (+0.2963)	0.2787 (+0.1407)	0.2882 (+0.1473)
EMMA-BBP in Less-manual	0.6372 (+0.3211)	0.2900 (+0.1520)	0.2897 (+0.1488)

Compared to EMMA, EMMA in Less-manual with obfuscated false information removed has some performance improvement, mainly in the unseen environment. Obfuscated incorrect information affects the model’s ability to connect entities with text. For the EMMA-BBP in the All-manual framework, which contains a behavioral prediction module, the introduction of the behavioral prediction module enables the combination of entities and behavioral information to eliminate the problem of ambiguity of the same entities in the text due to motions. Although the improvement of EMMA-BBP over EMMA-BBP in All-manual is inconspicuous, this is also due to the MESSENGER environment. Since the entities in this environment act differently from the textual descriptions in the required rounds, the model is misled to make incorrect behavioral judgments. In contrast, EMMA-BBP in Less-manual is an idealized framework of EMMA-BBP for text-matching module. The performance of this framework demonstrates that eliminating confusing text information can enhance the model’s generalization ability in an unseen environment.

Through comparative experiments involving EMMA-BBP and other baseline models, we have arrived at the following conclusions: (a) Through utilizing the behavior prediction module for labeling entities, agents can gain insights into their movement patterns. This obviously helps to enhance the model's generalization capabilities. (b) The elimination of interference information contributes to the transfer of policies learned by agents in known environments to unseen ones, enhancing their generalization capabilities. (c) While the win rate in an unseen environment remains below that of humans [9], we attribute this disparity to the behavioral prediction module. Our behavioral labels heavily rely on the entity’s actions throughout the 4 rounds. In cases where the behavior actions do not align with the textual description, our model’s performance is adversely affected.

## Limitation and Discussion

We believe that it is difficult for agents to learn the behavior in text descriptions solely through language information. To enhance the agents’ ability to learn from text and adapt to unseen environments, we label entities behavior. We also eliminate redundant text information to improve the agents’ generalization ability in unseen environments. In our model, we indicate the motion behavior of the entities in the form of labels. These labels are not directly given to the agents but are determined by the agents’ ability to think and judge like humans. Our approach is of great significance in promoting RL using natural language. By supplementing the description of motion information, agents can easily eliminate ambiguity caused by motion information. Our text-matching module also provides some inspiration for exploring language-conditioned RL. Statements with the same meaning often have different forms of expression. The introduction of text matching is able to transform semantically identical statements into a unified expression form, thereby enhancing the agents’ understanding of semantics. Although our model is designed for grid environments, it is able to be extended to more complex visual input situations. For example, entity information in images can be extracted through pretrained visual networks and then converted into text information for input into our framework. In the face of real-world scenarios with complex dynamics, our framework can consider expanding the fusion of sensors. By utilizing sensor information, agents can better learn the relationship between motion and text. However, our method relies on stationary agents to assess the behavior of entities, which may be time-consuming during training. Additionally, our model still needs to address the challenge of deducing entity behavior solely from textual information. We acknowledge these challenges and plan to address them in future work.

## Conclusion and Future Work

In this paper, we propose the EMMA-BBP framework based on EMMA. This framework includes behavioral prediction and text-matching modules, where the behavioral prediction module adds behavior information to the entities in the third stage of MESSENGER, thereby improving the model’s generalization ability. The text-matching module removes interference information from the original text based on entity behavior. It improves the generalization ability of the model to a certain extent. We test the EMMA-BBP framework on the training, test, and validation sets of MESSENGER stage 3. The results indicate that our framework improves EMMA’s performance. Our EMMA-BBP utilizes behavioral prediction and text-matching modules to predict entity behavior while filtering out interference information accurately.

In future work, we need to further optimize the issues of time-consuming behavioral prediction of the module and the inability of agents to associate actions with text. We face the following challenges. (a) Establishing a direct connection between text and actions: Consider incorporating attention to action text into language-conditional policies to enable agents to infer entities’ actions based on text information. (b) Optimization of the framework: One of the reasons for the current long-time consumption is due to defects in the MESSENGER environment, which requires considering more rounds to label entities’ behaviors. We will continue to explore possible paths and potential solutions to address the above challenges.

## Data Availability

Data and code will be available upon request.
